# Regional and size-dependent effects of ambient agricultural particulate matter on Ah-receptor activity and inflammatory responses in human U937 macrophages

**DOI:** 10.1080/08958378.2026.2676722

**Published:** 2026-06-11

**Authors:** Xiyuan Li, Jiang Chengcheng, Chuanzhen Zhang, Qianqian Lin, Savannah M. D’Evelyn, Toby Clark, Keith J. Bein, Qi Zhang, Alyssa Lundberg, Sarah Kado, Jack C. Connolly, Thomas Haarmann-Stemmann, Christopher R. Niedek, Yi He, Wei Li, Kent E. Pinkerton, Christoph F. A. Vogel

**Affiliations:** a School of Control Science and Engineering, Shandong University, Jinan, China;; b Center for Health and the Environment, University of California, Davis, California, USA;; c Memorial Sloan Kettering Cancer Center, BCMB Allied Program, Weill Cornell Graduate School of Medical Sciences, New York, New York, USA;; d Department of Gastroenterology, the First Affiliated Hospital of Shandong First Medical University & Shandong Provincial Qianfoshan Hospital, Jinan, China;; e Air Quality Research Center, University of California, Davis, California, USA;; f Department of Environmental Toxicology, University of California, Davis, California, USA;; g Leibniz Research Institute for Environmental Medicine, Düsseldorf, Germany

**Keywords:** Particulate matter, inflammation, macrophages, AhR, NF-κB

## Abstract

**Aims:**

Size-fractionated ambient particulate matter (PM) was collected from two of the most highly PM-polluted agricultural regions in California the Imperial Valley (PMIV), and San Joaquin Valley (Parlier, PMPA), to compare the effects of particle source, size, and duration of exposure on inflammatory gene expression, cell viability, and aryl hydrocarbon receptor (AhR) activation.

**Methods:**

Here we tested that the chemical composition of the PM would provoke different effects unique to the PM size fraction and with an association between exposure time, activation of AhR, and the expression of inflammatory genes. Human U937-derived macrophages were used to measure inflammatory biomarkers, cell viability and engulfment of PM. Particles across three size fractions – ultrafine (≥ 0.1 μm), fine (0.1–2.5 μm), and coarse (2.5–10 μm) were tested.

**Results:**

Gene expression varied by PM source, size, and duration of exposure. PMIV typically induced a greater level of gene expression than PMPA of the same size fraction. For the 12-h experiments, ultrafine and coarse particle fractions were the most potent stimulators of gene expression compared to the control, irrespective of PM source. The results show that ultrafine/fine PMIV and fine PMPA typically produced the greatest increase in mRNA levels compared to the control in an AhR-dependent manner.

**Conclusions:**

Ultrafine and fine PM from both sites (PMIV and PMPA) preferentially engaged AhR-dependent signaling, whereas coarse PM activated NF-κB–mediated inflammatory pathways. Overall, this study demonstrates PM size- and time-dependent effects on inflammatory gene expression and highlights a distinction between AhR- and NF-κB–driven responses across PM fractions.

## Background

Ambient airborne particulate matter (PM) has been considered a serious health hazard for decades. PM plays a significant role in air pollution because PM can remain suspended in the air for prolonged periods of time and contain mixtures of chemicals that contribute to adverse health effects. There is growing evidence that exposure to PM has a significant effect on cardiopulmonary morbidity in humans, such as asthma and chronic obstructive pulmonary disease (COPD) (Fiordelisi et al. 2017; Losacco and Perillo 2018), as well as a heightened risk for mortality (Chen and Hoek 2020). In addition, exposure to PM can provoke neural disorders and inflammation in the human central nervous system (Liu et al. 2018; Shou et al. 2019). PM exposure can also increase the risk of stroke, trigger carotid artery disease, and affect cognitive function, linked to Alzheimer’s disease and other dementias (Babadjouni et al. 2017; Kilian and Kitazawa 2018).

PM may have different effects on health depending upon the chemical composition, size fraction, and exposure duration. In relation to size fraction, PM can be classified according to size or diameter (*d*) as ultrafine PM (*d* ≤ 0.17 μm; PM_0.1_), fine PM (0.17 μm <*d* ≤ 2.5 μm; PM_2.5_; including submicron fine (0.17 μm < *d* ≤ 1 μm and PMM_1-0.17_) and supermicron fine (1 μm < *d* ≤ 2.5 μm, PM_2.5-1_)), and coarse PM (2.5 μm < *d* ≤ 10 μm; PM_10_). Differences in observed health effects may be due to the fractional deposition of each particle size in the respiratory tract and lungs (Saxton 1996). Numerous epidemiological studies (Pattey and Qiu 2012; Bu et al. 2021) have indicated a strong correlation between PM_2.5_ and health, while toxicological studies have suggested greater risks with increased exposure to PM_0.1_ (Losacco and Perillo 2018; Byrne and Baugh 2008; Patten et al. 2021; Patten et al. 2020). It is also important to consider the impact of coarse particles, which should not be underestimated or dismissed within the complex mixture of ambient PM. PM_10_ can be deposited in the nasal cavity and lungs. The larger PM_10_ particles are found primarily in the upper respiratory tract during nasal inhalation and frequently in the lungs due to mouth-breathing (e.g. when a person is exercising or has a stuffy nose) (Churg et al. 2005; Cachon et al. 2014; Kang et al. 2015; Medina-Ramόn et al. 2006). Mouth-breathing bypasses filtration mechanisms in the nose, dramatically increasing PM_10_ deposition in the airways and lung parenchyma. Given PM_10_ also constitutes a large proportion of the total mass of PM pollutants, it is an essential size fraction to study. Despite the increasing evidence of the health risks of different size fractions of PM, few studies have directly compared the effects of different PM size fractions from the same location or source (D’Evelyn et al. 2023). Studying the impacts of different PM size fractions from the same source on a single cell type could help identify unique PM-size-dependent effects.

Another challenge in determining specific health effects of PM exposure is the use of one or two independent variables (e.g. exposure dose/concentration, or PM size) to investigate a limited number of outcomes. Thus, the cumulative body of knowledge is clouded by disparate results influenced by inter-laboratory and inter-study differences in methodology. In addition, most of the current studies on PM compare samples from the same region. Given that these studies lack comparative analyses of different regions, there is a benefit of completing such studies.

Therefore, the present study was designed to investigate the chemical composition of three different size fractions of PM to determine biological responses. PM was collected in the agriculturally-rich regions of Calipatria (Imperial Valley) and Parlier (San Joaquin Valley), California, which have high ambient PM levels (American Lung Association 2022). The Imperial Valley is located in the southeastern portion of the state, bordering the city of Mexicali, Mexico, and its predominant industry is agricultural. This county is consistently ranked as having one of the highest annual PM levels and rate of childhood asthma in California. These high PM levels may be due to the soil disturbance from heavy winds, human activities, traffic at the border, agriculture, unpaved roads, and movement of air pollution from the industrial city of Mexicali, which has a population of approximately one million residents (Watson and Chow 2001; Mendoza et al. 2010; Harnly et al. 2012). Additionally, the Salton Sea is located in the Imperial Valley. The Salton Sea is the second largest body of water in California. Since its formation by water diverted from the Colorado River in 1905, the lake has been increasingly polluted by agricultural runoff. In addition, the lack of fresh water input and the slowly shrinking lake size has exposed an increasingly dry shoreline and a significant source of PM with wind-blown dust (D’Evelyn et al. 2023). In the San Joaquin Valley, the ambient air quality, especially the level of PM, is a primary concern for the workers and residents there. High PM levels are predominantly due to agricultural practices including crop harvesting, burning, soil tilling, and diesel-powered water pumping. Other potential sources include emissions from dairy operations and highway vehicles, animal feed, and dried manure. PM levels in the California Central Valley—including Fresno County—are the highest recorded in the US (Ngo et al. 2010).

Using four different exposure scenarios, each of the PM size fractions were examined *in vitro* to compare their impact on gene expression for several inflammatory markers, as well as the activation of cytoplasmic aryl hydrocarbon receptor (AhR), which is critical to understanding some of the physiological and pathological effects of PM exposure. The organic fraction of PM may contain toxicants such as polycyclic aromatic hydrocarbons (PAHs) including benzo[a]pyrene (BaP) which have been identified as ligands of the AhR, a ligand-activated transcription factor that regulates multiple target genes including cytochrome P450 1a1 (CYP1A1) and inflammatory cytokines such as interleukin (IL)-1β and IL-8 (9). Exposure to environmental pollutants and activation of AhR signaling have been identified as risk factors to promote chronic inflammatory diseases and the development of cancer (10–14). Furthermore, studies from our group and others indicate that AhR plays a key role as an immunomodulator and as a mediator of toxic responses induced by particulate matter (PM).

We hypothesize that differences in the chemical composition of PM from the Imperial and San Joaquin Valleys would provoke different inflammatory effects unique to each size fraction and region. We anticipate that the PM fraction with a higher content of PAHs which may serve as potential AhR ligands would elicit greater effects on AhR-regulated genes including CYP1a1, COX-2 and IL-8. In addition, we hypothesize a positive association with activation of AhR and the expression of inflammatory genes depending on PM size, the source of PM and exposure time. For instance, the PM10 fraction is expected to enhance expression of inflammatory markers after longer exposure durations where the chemical components are less bioavailable and may require more time.

To our knowledge, the present study is the first to comprehensively compare the biological responses of PM from the Imperial and San Joaquin Valleys by considering the effects of PM size, source, exposure duration, and chemical composition. Our findings provide valuable insights into the impacts of source- and size-specific PM with varying exposure times.

## Materials and methods

### Reagents

Dimethyl sulfoxide (DMSO) was purchased from Sigma. 2,3,7,8-tetrachlorodibenzo-p-dioxin (TCDD) (>99% purity) was originally obtained from Dow Chemical Co. (Midland, MI, USA). 12-O-tetradecanoylphorbol-13-acetate (TPA) and other molecular biological reagents were purchased from Cayman Chemicals (Ann Arbor, MI, USA) and Applied Biosystems (Foster City, CA, USA). Toll-like receptor (TLR) ligand LPS was purchased from InvivoGen (San Diego, CA, USA).

### PM collection

PM sampling was performed in the agricultural communities of Imperial Valley and San Joaquin Valley in California in 2018. Samples from Imperial Valley were collected in Calipatria, California. These same PM samples from Imperial Valley were used to measure the toxicity of size fractioned particles *in vitro* and *in vivo* (D’Evelyn et al. 2023; D’Evelyn et al. 2021).

Samples from San Joaquin Valley were collected in Parlier (Fresno County), California. Ambient PM was collected in Imperial Valley during the summer (June to August, 2018) and in Parlier during the fall (October to November, 2018). No major regional wildfire activity during the collection period has been reported. The Imperial Valley sampling site was located on the grounds of the Calipatria School District, directly south of the Salton Sea. The San Joaquin Valley sampling site was situated at the Kearney Agricultural Research and Extension Center (KARE).

PM in Calipatria and Parlier were collected by deploying a fully remote-controllable PM Sampling and Measurement Platform (PMSAMP; Supplementary Figure S1), allowing the collection of size-segregated PM samples and real-time air quality and meteorological data. Ambient air was drawn through a 10-foot-tall, 8-inch-diameter sampling stack at the trailer’s top. The stack was plumbed through the roof and wall of the PMSAMP trailer into the ChemVol (CV) manifold, containing 10 separate stacks in the interior of the PMSAMP (Supplementary Figure S1). All PM samples from both regions were collected using CV samplers (Demokritou et al. 2002). operated at a high flow rate (900 liters/minute) and equipped with stacked impactor stages. In addition to providing after-filter support, each of the CV stacks had 0.17-, 1.0-, and 2.5-μm stages that could be combined in series to collect size-aggregated PM for a range of size cuts. These 10 CV stacks collected PM_0.1_, PM_1-0.17_, and PM_2.5-1_ fractions. One 10-μm stage was located upstream from the CV manifold to collect PM_10_. All PM size fractions, except ultrafine PM, were collected onto polyurethane foam (PUF) substrates. The ultrafine PM was collected onto Teflon-bound borosilicate glass microfiber filters (Pall Corporation, TX40H120WW-8 × 10).

### PM extraction

All particle extractions for sample preparations were conducted at the University of California, Davis. Previously described multi-solvent sonication extraction methods (Bein and Wexler 2014; Bein and Wexler 2015). were used based on the media for particle extraction. In brief, PM_0.1_ samples collected on glass microfiber filters underwent serial sonication in dichloromethane (DCM) followed by methanol (MeOH). The extract solutions were then filtered *via* 0.2 μm pore size syringe filters to remove contaminant filter material. Bulk solvent was removed *via* distillation, and the DCM and MeOH extracts were combined prior to final solvent removal *via* nitrogen blowdown. PM_1-0.17_ and PM_2.5-1_ samples collected on PUF were sonicated in milli-Q water followed by liquid–liquid extraction in DCM and solvent phase separation *via* a separatory funnel. The water and DCM extract solutions were then filtered using 8 μm pore size filter paper in a Buchner funnel with a fritted class disk (size M: 10–15 μm pore size). Water was removed to dryness *via* lyophilization, bulk DCM was removed *via* distillation, and the DCM and water extracts were combined prior to final DCM removal *via* nitrogen blowdown. The exact mass of all dry PM extracts was determined by pre- and post-weighing the final storage vials *via* a micro-analytical balance (Mettler Toledo XP56, with 1 μg measurement accuracy). PM_10-2.5_ was scraped directly off the PUF surface with a sterilized metal scraper that had been washed with soap and rinsed with deionized water followed by methanol. The abbreviations PM_IV_ and PM_PA_ were used to represent the PM sample extracts collected from Imperial Valley and San Joaquin Valley, respectively. For extracts from a specific size fraction and location, e.g. PM_10_ from Imperial Valley, the abbreviation was PM_10-IV_.

### Chemical characterization of PM samples by soot particle aerosol mass spectrometry

Chemical characterization of the PM extracts was performed at the University of California, Davis. PM extracts were thawed to 4 °C, suspended in Milli-Q water at 0.1 μg/μL and water bath-sonicated for 20 min (min) at room temperature to dissolve water-soluble PM and completely resuspend water-insoluble black carbon. After sonication, samples were diluted by half to 0.05 μg/μL by adding 1 mL of Milli-Q water to 1 mL of each suspension. Two milliliters of each suspension were delivered to a constant output atomizer and continuously atomized using argon as the carrier gas. The resulting particles were then dried in a silica gel diffusion dryer. Soot Particle Aerosol Mass Spectrometry (SP-AMS), with a standard AMS equipped with a 1,064-nm intracavity laser vaporizer based on the Single Particle Soot Photometer (SP2) design, was used to measure the total organic PM mass and composition, including refractory black carbon (rBC), according to previously published protocols (Ge et al. 2014; Sun et al. 2010). The sensitivity of SP-AMS is greater than 140 carbon ions detected per picogram of rBC mass sampled, with a 3-standard deviation (σ) detection limit of <0.1 μg/m^3^ for an average 60 s (s) and a mass-specific ionization efficiency relative to particulate nitrate of 0.2 ± 0.1 (Onasch et al. 2012). The SP-AMS data analysis was performed using a standard AMS data analysis tool-kits (SQUIRREL v1.63H and PIKA 1.23H).

### Selection of inflammatory biomarkers

Cellular responses to PM exposure were examined by quantifying the gene expression messenger ribonucleic acid (mRNA) levels of various inflammatory biomarkers. All gene expression markers were chosen for their critical roles in PM-induced inflammation. Cytochrome P450 Family 1 Subfamily A Member 1 (*CYP1a1*) is of interest given its ability to metabolize exogenous and endogenous compounds into oncogenic derivatives related to cancer initiation and development. Additionally, *CYP1a1* expression is a helpful indicator in determining exposure to AhR ligands, including polycyclic aromatic hydrocarbons (PAHs), and AhR activity (Badal and Delgoda 2014). Cyclooxygenase 2 (*COX-2*) inhibition attenuates cellular inflammatory damage and suppresses the production of inflammatory factors (Bian et al. 2019). Interleukin-8 (*IL-8*), a chemokine that activates the C-X-C Motif Chemokine Receptor 2 (*CXCR2*), is a critical mediator of attracting neutrophils and triggering atherosclerosis (Wu et al. 2011). The proinflammatory cytokine interleukin-1 beta (*IL-1*β) is involved in immune responses to many diseases and may increase disease severity. Pharmacological blockage of *IL-1*β signaling is beneficial in certain autoimmune and auto-inflammatory diseases (Mendiola and Cardona 2018). Neutrophils require directional guidance to cross complex venous wall structures to enter inflamed tissues, and the migration of neutrophils therein is mediated by the continuous action of C-X-C Motif Chemokine Ligand 2 (*CXCL2*). Therefore, studying *CXCL2* mRNA expression would provide further insights into the severity of the inflammatory response (Girbl et al. 2018). Tumor necrosis factor-alpha (*TNF-α*) acts in inflammatory diseases of the lung in several ways, inducing the aggregation of inflammatory cells, stimulating the production of inflammatory mediators, and causing airway hyperresponsiveness (Malaviya et al. 2017).

### U937 cell culture

Human U937-derived macrophages were used as the primary cell type for examining inflammatory responses because macrophages are a first-line defense against the uptake of inhaled particles in the lungs and initiation of inflammation. The U937 monocytic cells were acquired from the American Tissue Culture Collection in Manassas, VA and maintained for 2 days in RPMI 1640 medium, 10% fetal bovine serum (FBS) (Gemini, Woodland, CA), supplemented with 4.5 g/L glucose. All cell culture materials listed above, except the 10% FBS, were purchased from GIBCO (Thermo Fisher Scientific, Waltham, MA, USA). Cell cultures were maintained at concentrations between 2 × 10^5^ and 2 × 10^6^ cells/mL in 10 cm tissue culture dishes. For differentiation into macrophages, U937 cells were treated with 3 μg/mL TPA and allowed to adhere for 2 days in a 5% carbon dioxide (CO_2_) tissue culture incubator at 37 °C. After incubation, the cells were maintained in TPA-free medium for treatment. The differentiation status was confirmed by an attached cell phenotype and increased expression of a β-galactoside-binding lectin, Galectin-3 (MAC-2), measured in quantitative real-time reverse transcription (qPCR) (Castañeda et al. 2018).

### Generation of CRISPR/Cas9 AhR mutants of U937 cells

CRISPR/Cas9-mediated *AhR* knockout U937 cells were generated as previously described for MCF-7 cells (Vogel and Matsumura 2009). Briefly, the CHOPCHOP CRISPR design tool (http://chopchop. cbu.uib.no/(accessed 21 March 2023)) was used to select a guide RNA targeting exon 2 of *AhR* (5′-AAGTCGGTCTCTATGCCGCTTGG-3′). The gRNA was cloned into the PX458 plasmid (Addgene #48138). U937 cells were transfected with the nuclease plasmid in antibiotic-free medium using FuGENE HD (Roche), following the manufacturer’s instructions. After 48 h, GFP-positive cells were isolated by FACS and seeded as single cells into 96-well plates. Clonal populations were screened by high-resolution melt analysis and confirmed by Sanger sequencing. *AhR* knockout was further validated using the DNA/RNA Shield^™^ kit (Zymo Research, Irvine, CA, USA).

### Cell viability assay

A trypan blue exclusion test was used to determine the dose-based effects of PM exposure on U937 macrophage viability to determine the optimal PM exposure concentration. Briefly, cells maintained in TPA-free medium were first exposed to various concentrations of each PM extract for 24 h. Then, an aliquot of cell suspension was centrifuged for 5 min at 400 g (relative centrifugal force) and the supernatant was discarded. The cell pellet was resuspended in 1 mL phosphate-buffered saline (PBS) and a 10 μL portion of the resuspended cell pellet was placed in 190 μL PBS with 200 μL trypan blue (0.5% dilution in 0.85% sodium chloride, NaCl) for 5 min. Afterward, 10 μL of the cell suspension was loaded into a hemocytometer, and the proportion of nonviable to viable cells was determined. The 10 μg/mL PM concentration was determined optimal because no cell death was detected when the aforementioned inflammatory biomarkers were measured.

### Cell exposure

Before cell exposure, the PM samples were bath sonicated for 25 min to minimize PM aggregation. The U937-derived macrophages were exposed to 10 μL PBS (solvent/negative control) or 10 μg/mL PM (PM_0.1-IV_, PM_2.5-IV_, PM_10-IV_, PM_0.1-PA_, PM_2.5-PA_, or PM_10-PA_) added directly to the media and incubated. Exposure times were set at 6, 12, and 24 h. Exposure durations between 4 and 24 h are most common for *in vitro* systems, as they enable insight into potential mechanisms driving toxic responses to PM (Cavanagh et al. 2009; Lauer et al. 2009). All assays were performed in triplicate for each treatment (3 wells/exposure treatment/duration time).

### Slide preparation

After treatment with PM, macrophages were prepared for imaging. 100 μL of the cell suspension was then loaded into a cytospinner (Thermo Scientific) using funnels (Fisherbrand) and cytospun at 1500 rpm for 10 min to evenly distribute them into a single layer on slides (Fisherbrand). The slides were then allowed to dry for 10 min protected from dust. Once they were dry, the cells were fixed for 20 min using a solution of 1% paraformaldehyde (Electron Microscopy Services) in PBS. The slides were then rinsed twice with PBS and once with double distilled water. After drying for at least two hours, #1 coverslips were mounted (Fisherbrand) using low viscosity resin mounting media (Fisherbrand) and allowed to cure overnight.

### Imaging

The slides were imaged using a CytoViva Hyperspectral Imaging system (CytoViva Inc.). This method images unstained slides using light at an oblique angle, causing metallic PM to appear reflective without needing to stain the cells. This property makes the CytoViva a highly effective option to visualize the uptake of PM in cells. Contaminants such as dust can appear reflective under the CytoViva, so each time a new batch of cells was imaged an untreated control sample was prepared and imaged to establish a baseline level of reflective PM present. Images were taken and exported using Ocular^™^ Image Acquisition Software (Qimaging).

### Quantification of inflammatory cellular responses

Zymo Research RNA isolation kits (Irvine, CA) were used to isolate total RNA from PM-exposed U937 macrophages. The RNA concentration was determined using NanoDrop Spectrophotometers (NDS) according to the manufacturer’s instructions. Complementary deoxyribonucleic acid (cDNA) synthesis was conducted using the High-Capacity cDNA Reverse Transcription Kit following the manufacturer’s instructions (Applied Biosystems, Foster City, CA) as described previously (Castañeda et al. 2018). Finally, qPCR was performed with β-actin as a housekeeping gene to quantify the mRNA expression of the selected biomarker genes (Section 2.4, above) (Chen et al. 2018). A Light Cycler Instrument (LC480 Roche Diagnostics, Mannheim, Germany) and a QuantiTect Fast SYBR Green PCR Kit (Qiagen) were used according to the manufacturers’ instructions. A melting curve analysis was then conducted on the PCR products to double-check the amplification specificity.

### Dioxin-responsive element (DRE) and NF-κB luciferase reporter assay

In the present study, stably pTX.Dir (a dioxin-responsive element (DRE)-luciferase reporter construct)-transfected HepG2 cells kindly provided by Berghard et al. (1993), were used to determine AhR activation and examine responses to the various PM samples (Vogel et al. 2007). The pTX.Dir plasmid construct contained two DRE sequences that span from nucleotides −1026 to −999 relative to the transcription start site of the rat *CYP1a1* gene. AhR binds to the DRE sequences, which were inserted into a vector containing the thymidine kinase promoter and a luciferase reporter gene of the herpes simplex virus. As a result, the bioluminescence of the luciferase directly corresponds to the potency of the test material (PM) in terms of activating the AhR and increasing *CYP1a1* mRNA. The nuclear factor kappa-light-chain-enhancer of activated B cells (NF-κB) reporter construct was transiently transfected into HepG2 cells as described previously (Vogel et al. 2007). The NF-κB luciferase reporter was a kind gift of Courtney Sulentic (Wright State University, OH). The plasmid DRE and NF-κB reporter constructs were amplified and purified with Zymo PURE-Endo Zero plasmid isolation kit (Zymo Research). AhR and NF-κB are present in lung cells and can be activated by specific PAHs (Chan et al. 2014; Hawk et al. 2002). To investigate whether PM_IV_ and PM_PA_ are capable of activating these pathways, HepG2 cells (ATCC HB-8065, Manassas, VA) were utilized, as their high transfection efficiency makes them well suited for detecting ligand-induced activation of AhR and NF-κB.

The transfected HepG2 cells were seeded onto 24-well plates, at a density of 1.2 × 10^4^ cells/well, in Dulbecco’s Modified Eagle Medium (Gibco Life Technologies, Grand Island, NY) containing 10% FBS, 100 units of penicillin, and 100 μg/mL streptomycin. Exposures were performed in triplicate for cells treated with 10 μL/mL PBS (negative control), 10 μg/mL resuspended PM extract (PM_0.1_, PM_2.5_, or PM_10_), or 1 nM TCDD and incubated at 37 °C for 6 h. The activation of the AhR and NF-κB transcription factors is an early event in the transcriptional regulation of the selected target genes which usually peaks at around 4 to 6 hrs after stimulation.

The prototypical AhR ligand TCDD and LPS served as positive controls. A luminometer (Berthold Lumat LB 9501/16, Pittsburg, PA) and 20 μL of cell lysate were used to measure chemiluminescence. Results were expressed as relative light units (RLU).

### Statistical analysis

The data were analyzed using GraphPad Prism 8.0 (GraphPad Software, Inc. La Jolla, CA). Four normality tests (Anderson-Darling, D’Agostino-Pearson omnibus, Shapiro-Wilk, and Kolmogorov-Smirnov) and one equal variance test (Bartlett’s test) were performed to ensure the data met the one-way analysis of variance (ANOVA) requirements. ANOVAs and post hoc Tukey’s tests were applied to analyze statistically significant differences between groups at a level of *p* < 0.05. The gene expression data were uniformly standardized to multiples of the relative change in the housekeeping β-actin gene and expressed as the mean ± standard error of the mean (SEM). The β-actin cytoskeletal protein has ubiquitous cellular functions in cell structure and motility (Sikand et al. 2012). Therefore, the data standardization practice served as an internal control for gene expression normalization. Since the reliance on a single housekeeping gene may represent a limitation, we have verified the stability of β-actin by testing additional housekeeping genes including GAPDH and rps13 as shown in supplemental Figure S3. No significant differences of the expression pattern was detected between the housekeeping genes and the treatment groups.

## Results

### Chemical characterization

**Figure 1.**
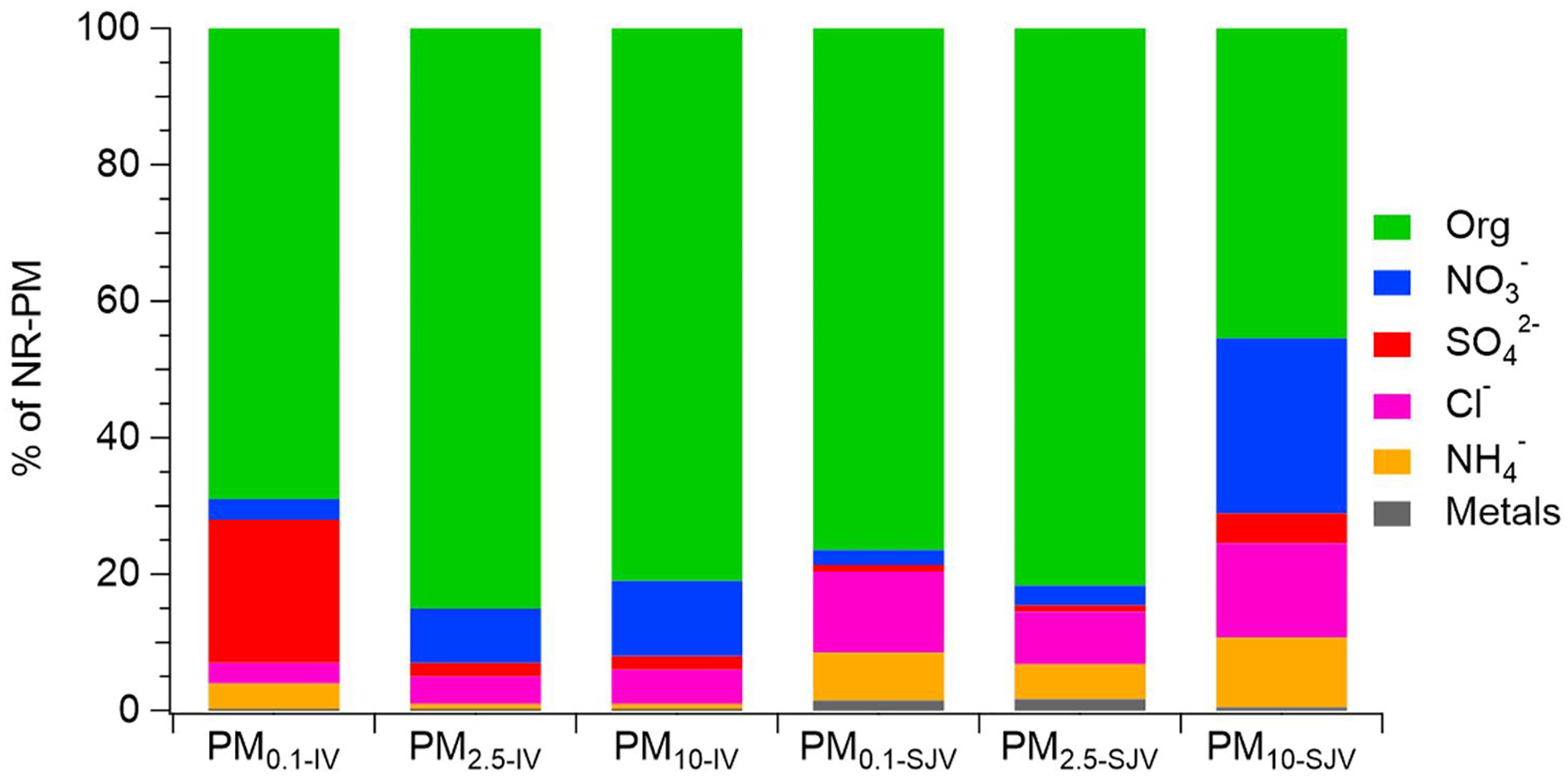
SP-AMS results for PM extracts from two regions in California. Imperial Valley (PM_IV_; first 3 bars) and San Joaquin Valley (PMSJV; bars 4,5, and 6) extracts were examined, but not total PM mass. (A-C), chemical composition of PM_0.1-IV_, PM_2.5-IV_, and PM_10-IV_ respectively. (D-F) Chemical composition of PM_0.1-PA_, PM_2.5-PA_, and PM_10-PA_, respectively. Each bar illustrates the proportions of organic and inorganic compounds in a specific PM extract. Two samples were run per extract type.

SP-AMS analysis showed that the main components of PM_IV_ and PM_PA_ were organic compounds (Figure 1). The percentages of nitrate (NO_3_^−^) and chlorine (Cl^−^) were increased gradually along with the particle sizes of PM_IV_, while the opposite trend was found in the relative abundances of sulfuric acids (SO_4_^−^) and amines (NH_4_^+^), especially for SO_4_^−^. However, no correlations were found in the chemical compositions of different sizes of PM_PA_, except for a positive correlation of NO_3_^−^ with the particle size.

For PM_0.1-PA_ and PM_2.5-PA_, the vast majority of the Cl^−^ signal was associated with NaCl. However, there were mass spectral indications of the presence of other chlorine-containing compounds in the samples (Supplementary Figure S2).

**Figure 2.**
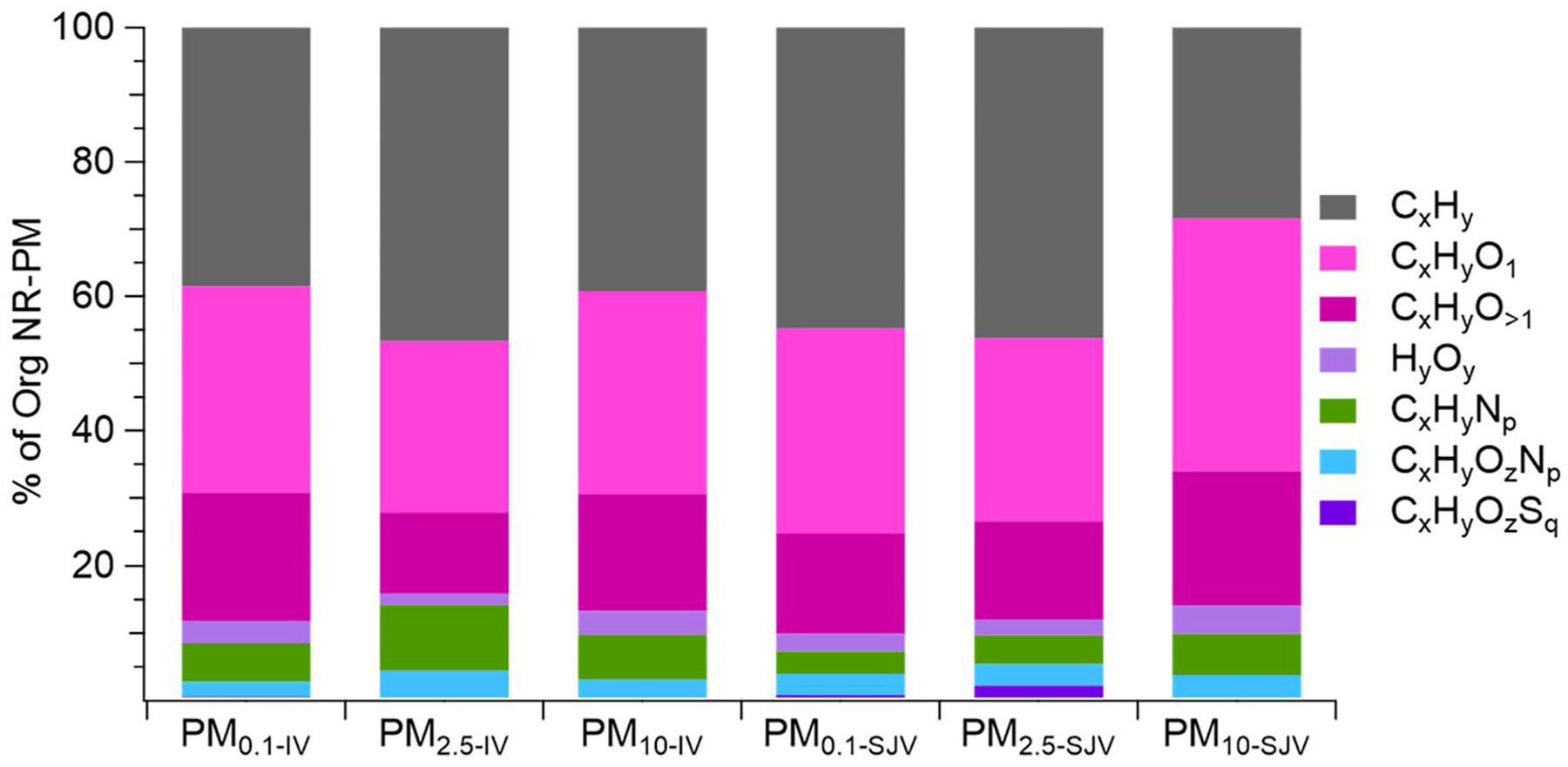
SP-AMS ion family results for PM extracts from two regions in California. Imperial Valley (PM_IV_; first 3 bars) and San Joaquin Valley (PMSJV; bars 4,5, and 6) extracts were examined. (A-C) Chemical composition of PM_0.1-IV_, PM_2.5-IV_, PM_10-IV_, respectively. (D-F) Chemical composition of PM_0.1-PA_, PM_2.5-PA_, and PM_10-PA_, respectively. Each bar illustrates the proportions of hydrocarbon and oxygenated hydrocarbon compounds in a specific PM extract. Two samples were run per extract type.

Results of the organic chemical composition analysis of all PM samples are shown in Figure 2(A–F). All PM_IV_ and PM_PA_ size fractions were predominantly hydrocarbons (Figure 2(A–C) and 2(D–F), respectively). When compared to other size fractions from the same region, PM_10-PA_ (Figure 2(f)) appeared to have the highest proportions of oxygenated hydrocarbons. However, hydrocarbons were not the most abundant in PM_IV._

### PM uptake and engulfment

**Figure 3.**
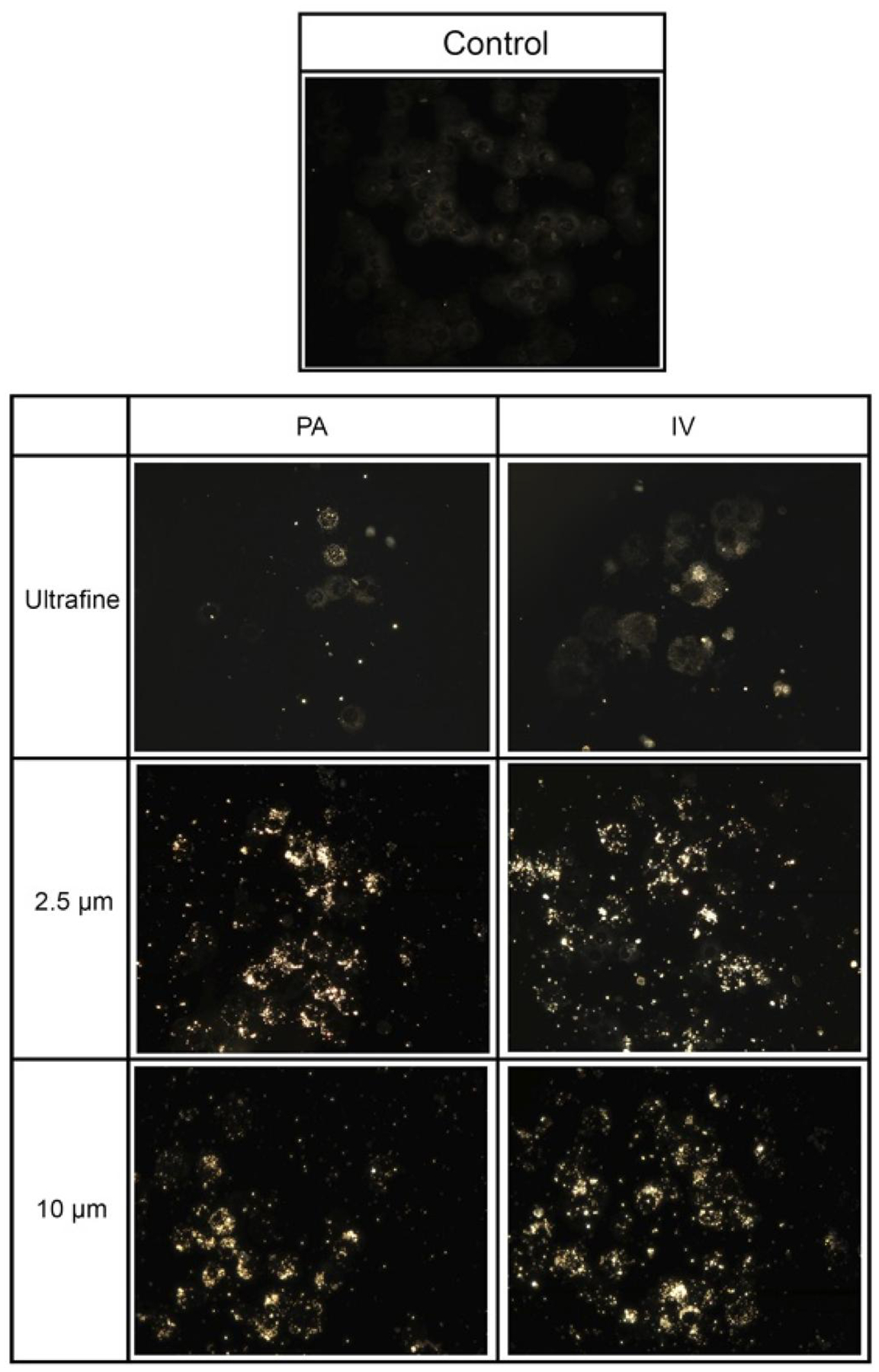
The imaging CytoViva microscope shows the uptake of PM_0.1-IV_, PM_2.5-IV_, PM_10-IV_ and the PM_0.1-PA_, PM_2.5-PA_, and PM_10-PA_ including a control sample of untreated cells. U937-derived macrophages were treated for 24 h with 10 μg/mL of PM_0.1_, PM_2.5_, or PM_10_ from imperial Valley or parlier.

The imaging results obtained using the CytoViva hyperspectral microscope illustrate the cellular uptake and distribution of PM samples of different aerodynamic diameters—PM_0_._1_-IV, PM_2_._5_-IV, and PM_10_-IV—as well as their corresponding PA-labeled variants, PM_0_._1_-PA, PM_2_._5_-PA, and PM_10_-PA (Figure 3). Each of these samples was compared against a control group of untreated cells to assess background signal and confirm that observed spectral signatures originated from particle internalization rather than intrinsic cellular components. The use of the CytoViva microscope was intended as a qualitative confirmation of PM–cell association and cellular PM uptake rather than a detailed compositional or quantitative assessment of the PM. The CytoViva images reveal differences in particle accumulation patterns, intensity, and localization depending on particle size and composition, providing visual evidence of how nanoscale and microscale particulates interact with and penetrate cellular structures.

### Cell viability

**Figure 4.**
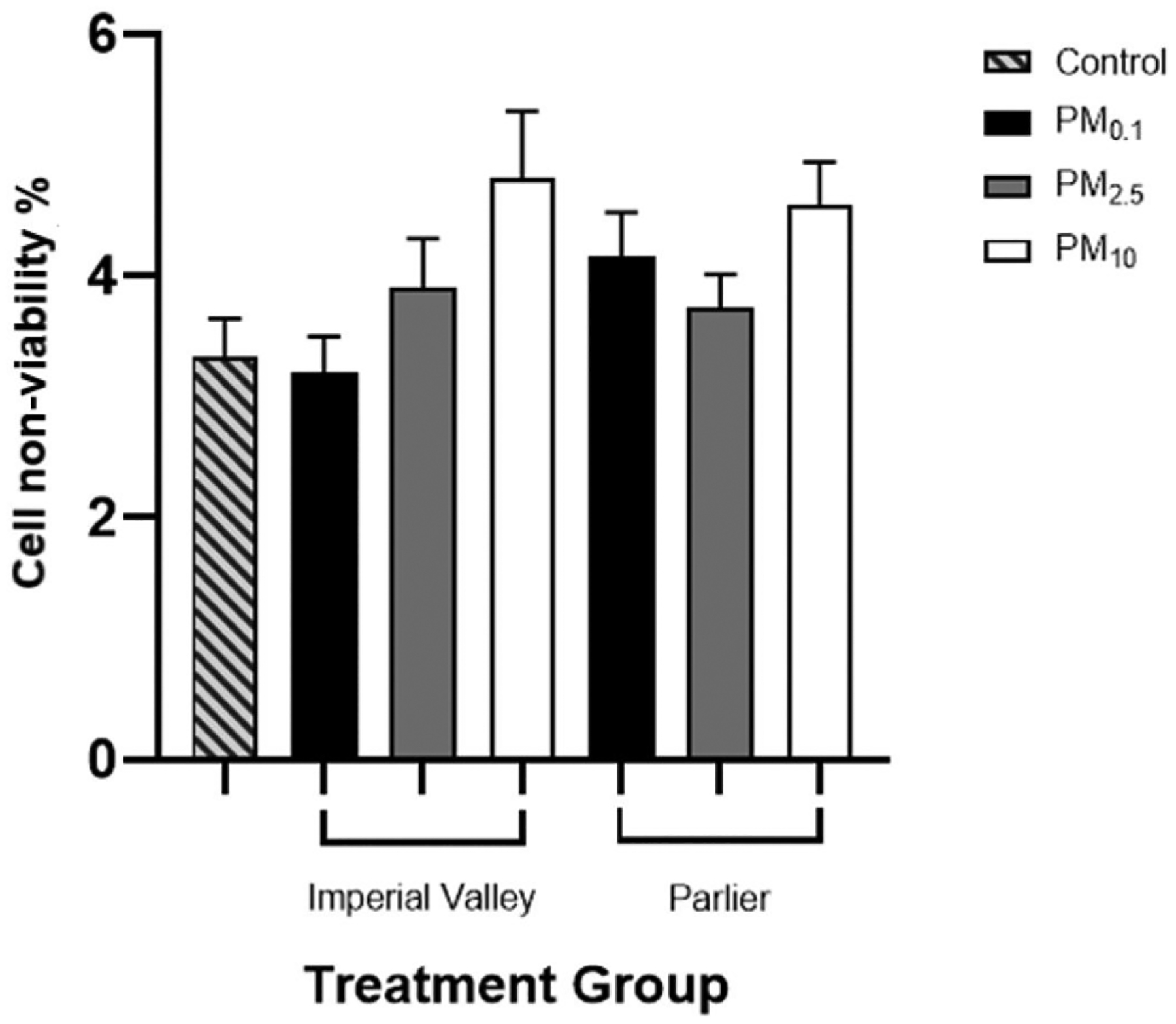
Effect of imperial Valley and San Joaquin Valley (Parlier) PM extracts on cell viability. Human U937-derived macrophages were treated with 10 μg/mL PM_0.1_, PM_2.5_, or PM_10_ for 24 h, and cell viability was tested *via* trypan blue exclusion tests. The graph shows the mean ± standard error of the mean (SEM) for each treatment. The effect of PM size on cell viability was tested *via* one-way ANOVA for each location.

24 h exposure to 10 μg/mL of PM_0.1_, PM_2.5_, or PM_10_ from Imperial Valley or San Joaquin Valley (Parlier) was found to not have a statistically significant effect on the viability of U937-derived macrophages when compared to PBS (*p* > 0.05; Figure 4). Thus, 10 μg/mL with minimal cell death was chosen to examine gene expression in all experiments.

### Effects of PM source on gene expression

**Figure 5.**
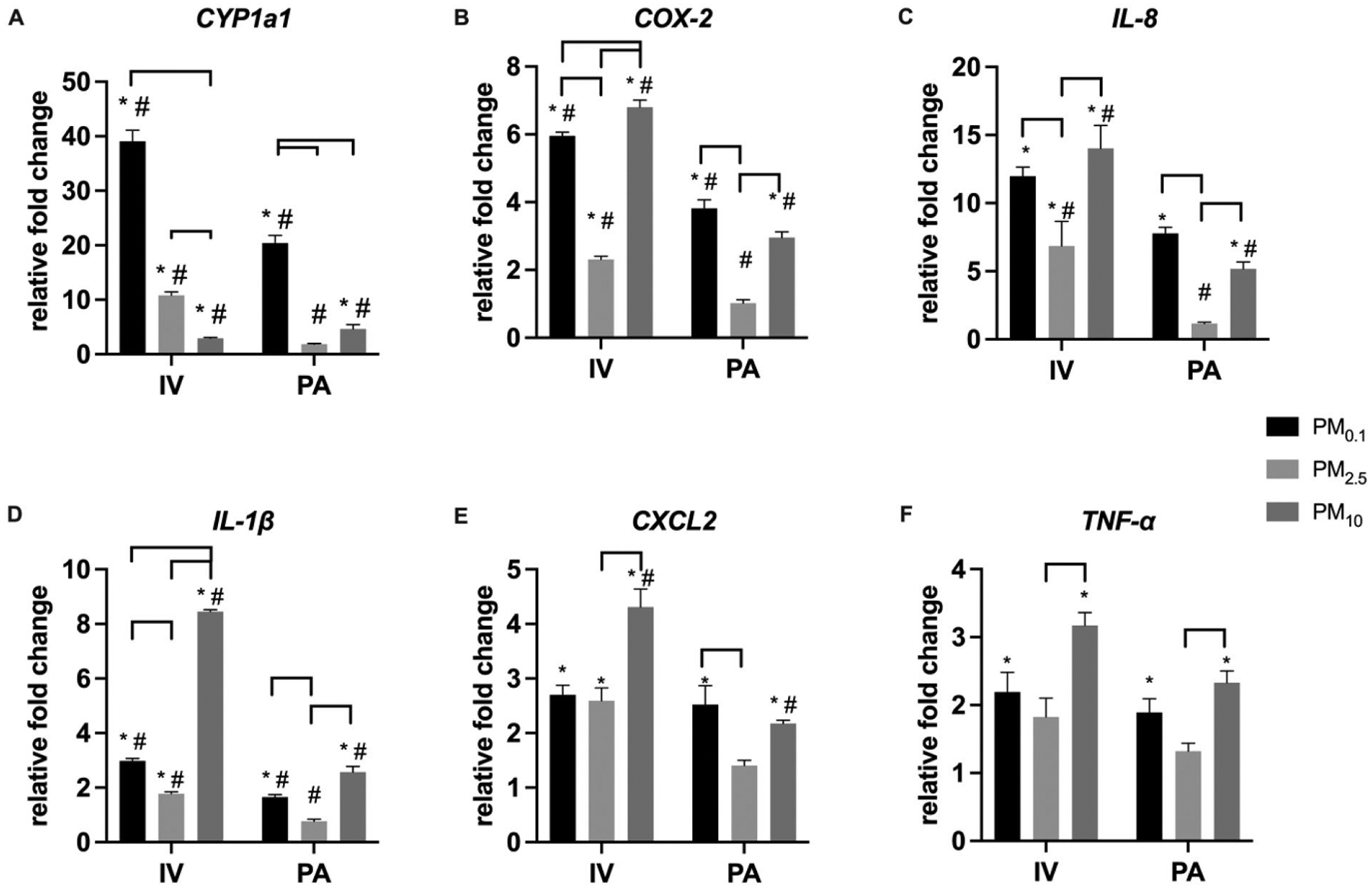
Gene expression profile after 12-h incubation of U937 macrophages with PM extracts. Gene expression was measured, normalized to that of the β-actin housekeeping gene, and graphed as fold changes relative to the phosphate-buffered saline (PBS) control. Fold changes are shown on the y-axes as functions of exposure to different size fractions of particulate matter (PM) extracts from Imperial Valley (IV) and San Joaquin Valley (PA). Statistical analyses were performed using one-way ANOVAs and post hoc tukey’s tests. Asterisks (*) denote statistically significant (*p* < 0.05) differences from PBS exposure. Hashes (#) indicate statistically significant (*p* < 0.05) differences between size-matched extracts from different sources. Brackets indicate statistically significant (*p* < 0.05) differences between size fractions from the same source.

Size-matched PM_IV_ and PM_PA_ revealed the following: (1) PM_0.1-IV_ and PM_2.5-IV_ significantly increased the expression of *CYP1a1* (Figure 5(A)), *COX-2* (Figure 5(B)), and *IL-1*β (Figure 5(D)) relative to PM_0.1-PA_ (*p* = 0.0018, *p* = 0.0015, and *p* = 0.0005, respectively) and PM_2.5-PA_ (*p* = 0.0002, *p* = 0.0007, and *p* = 0.0005, respectively); (2) PM_2.5-IV_ significantly increased the expression of *IL-8* relative to PM_2.5-PA_ (*p* = 0.037; Figure 5(C)); and (3) PM_10-IV_ significantly increased the expression of all biomarker genes, except *CYP1a1* and *TNF*-α, relative to PM_10-PA_ (Figure 5(B–E); *p* = 0.0001 for *COX-2*; *p* = 0.0072 for *IL-8*; *p* < 0.0001 for *IL-1*β; *p* = 0.0030 for *CXCL2*). There was a statistically significant increase in PM_10-PA_- versus PM_10_-_IV_-induced *CYP1A1* mRNA (*p* = 0.0110), but no significant trend was observed for an increased *TNF*-α mRNA in PM_10-IV_- versus PM_10-PA_-exposed cells. Still, cumulative results suggested PM_IV_ was generally more inflammatory than PM_PA_ irrespective of the tested size fraction (Figure 5(B–E)); PM_IV_-induced expression of *COX-2*, *IL-8*, *IL-1*β, and *CXCL2* was typically >1.5-times that provoked by size-matched PM_PA_ extracts (Figure 5(B–E)).

### Effects of PM size on gene expression (12-h exposure)

PM_IV_ samples revealed statistically significant differences compared with PM_10-IV_-induced gene expression for PBS, PM_2.5-IV_, and PM_0.1-IV_ exposure for 12 h (Figure 5(B–F)). The 12-h potency of PM_10-IV_ was such that mRNA levels for all the measured biomarker genes were increased relative to PBS (Figure 5(A–F)). PM_10-IV_ exposure also resulted in a statistically significant increased expression of all inflammatory markers except *CYP1a1* relative to PM_2.5-IV_ (*p* < 0.0001 for *COX-2*, *p* = 0.0431 for *IL-8*, *p* < 0.0001 for *IL-1*β, *p* = 0.0129 for *CXCL2*, and *p* = 0.0159 for *TNF*-α; Figure 5(B–F)), as well as *COX-2* and *IL-1*β compared to PM_0.1-IV_ (*p* = 0.0218 and *p* < 0.0001, Figure 5(B,D), respectively). PM_0.1-IV_ was similar to PM_10-IV_ to enhance mRNA expression for all genes compared to the PBS control. PM_0.1-IV_ also increased the expression of all biomarker genes except *CYP1a1*, *CXCL2,* and *TNF*-α compared to PM_2.5-IV_ (*p* < 0.0001 for COX-2, *p* = 0.0451 for IL-8, and *p* = 0.0004 for *IL-1*β; Figure 5(A,E,F), respectively) and increased *CYP1a1* expression compared to PM_10-IV_ (*p* < 0.0001; Figure 5(A)).

Of the PM_PA_ samples, the potencies of PM_0.1-PA_ and PM_10-PA_ were similar, with one or both producing significant elevations in gene expression compared to PBS and PM_2.5-PA_ (Figure 5(B–F) after 12-h exposure. The only statistically significant difference between PM_0.1-PA_ and PM_10-PA_ was the expression of *CYP1a1* mRNA (*p* = 0.0007), a 4-folder higher increase (*p* < 0.05) with exposure to PM_0.1-PA_ compared to PM_10-PA_ (Figure 5(A)).

### Effects of exposure duration on gene expression

**Figure 6.**
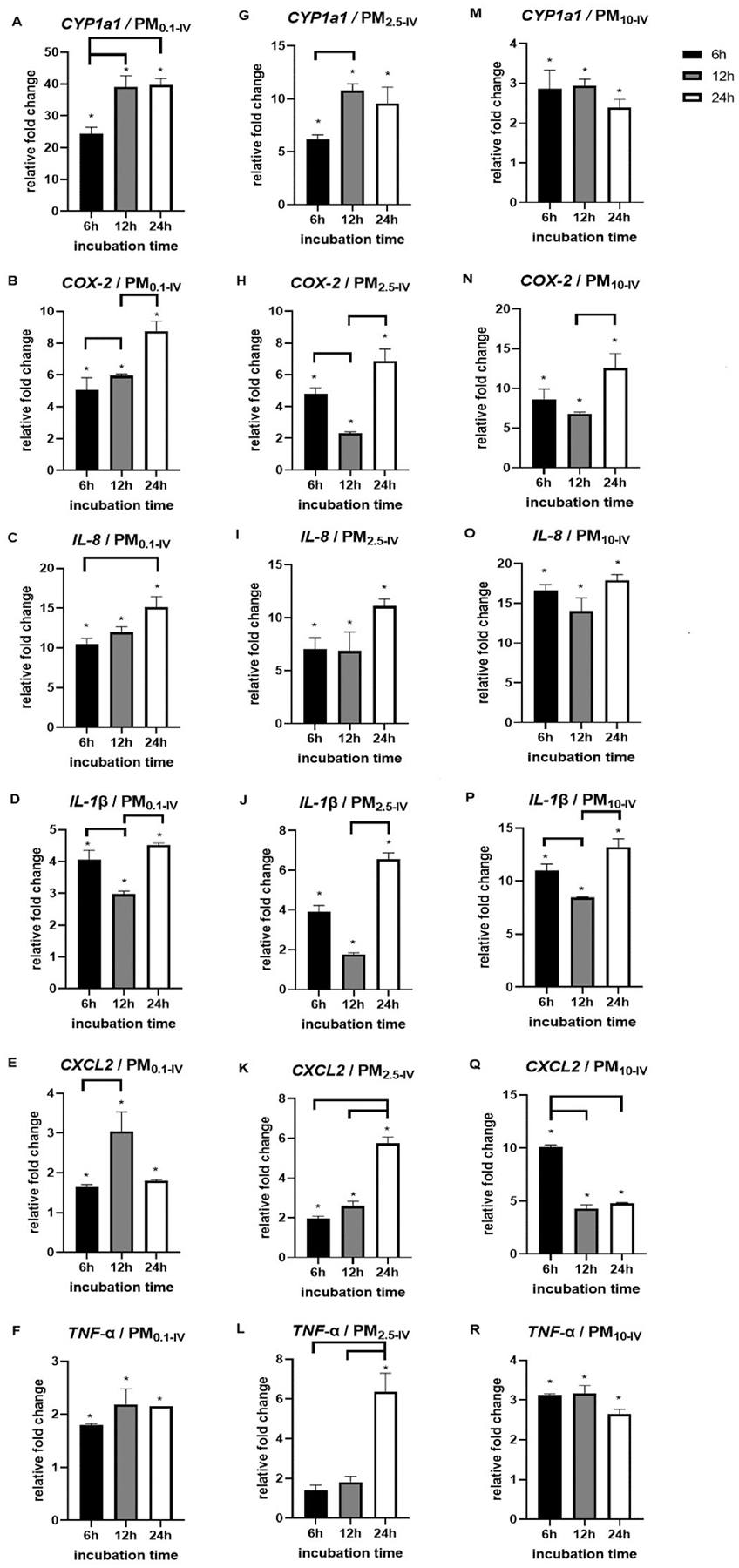
Gene expression profile in U937 macrophages after variable-length exposures to PM_IV_ versus PBS. Gene expression was measured, normalized to that of the β-actin housekeeping gene, and graphed as fold changes relative to the PBS control. Fold changes are shown on the y-axes as functions of 6-, 12-, or 24-h exposure to different size fractions of particulate matter (PM) extracts from Imperial Valley (IV), California. Panels A–F, G–I, and M–R represent exposure to PM_0.1-IV_, PM_2.5-IV_, and PM_10-IV_, respectively. Statistical analyses were performed using one-way ANOVAs and post hoc tukey’s tests. Asterisks (*) denote statistically significant (*p* < 0.05) differences from PBS exposure. Brackets indicate statistically significant (*p* < 0.05) differences between size fractions from the same source.

Overall, there was no consistent association between exposure duration and gene expression. When considering PM_IV_ exposures alone, nearly all scenarios produced statistically significant increases in gene expression compared to PBS. The only exceptions were 6- and 12-h exposure to PM_2.5-IV_ with 2-fold increase in *TNF-α* relative to PBS; however, this difference was not statistically significant. Both significant (*p* < 0.05) and non-significant linear increases in mRNA were observed with increasing exposure duration (Figure 6(A–C,F,I,K–I) in many PM_IV_-exposed cell groups. These responses suggest the duration of PM_IV_ exposure could be positively associated with increases in the expression of inflammatory genes. However, the majority of other PM_IV_-exposed groups exhibited a non-monotonic (U- or inverted U-shaped) response pattern (Figure 6(D–E,H,J,N-P)), with mRNA expression markedly higher or lower following 12-h exposure compared to the 6- and 24-h exposure. A consistent U-shaped response pattern was observed for *IL-1*β expression for all PM_IV_ size fractions. Overall, responses to PM_0.1-IV_ and PM_2.5-IV_ were increased with longer exposure compared to PM_10-IV_. This finding can be observed in the number of statistically significant increases observed for longer versus shorter exposure duration, indicating that exposure duration could be an important factor in the *in vitro* activity of the PM_IV-0.1_ and PM_IV-2.5_ fractions. In contrast, an inverse relationship for PM_10_-_IV_ exposure duration and *CXCL2* gene expression was observed (Figure 6(Q)).

**Figure 7.**
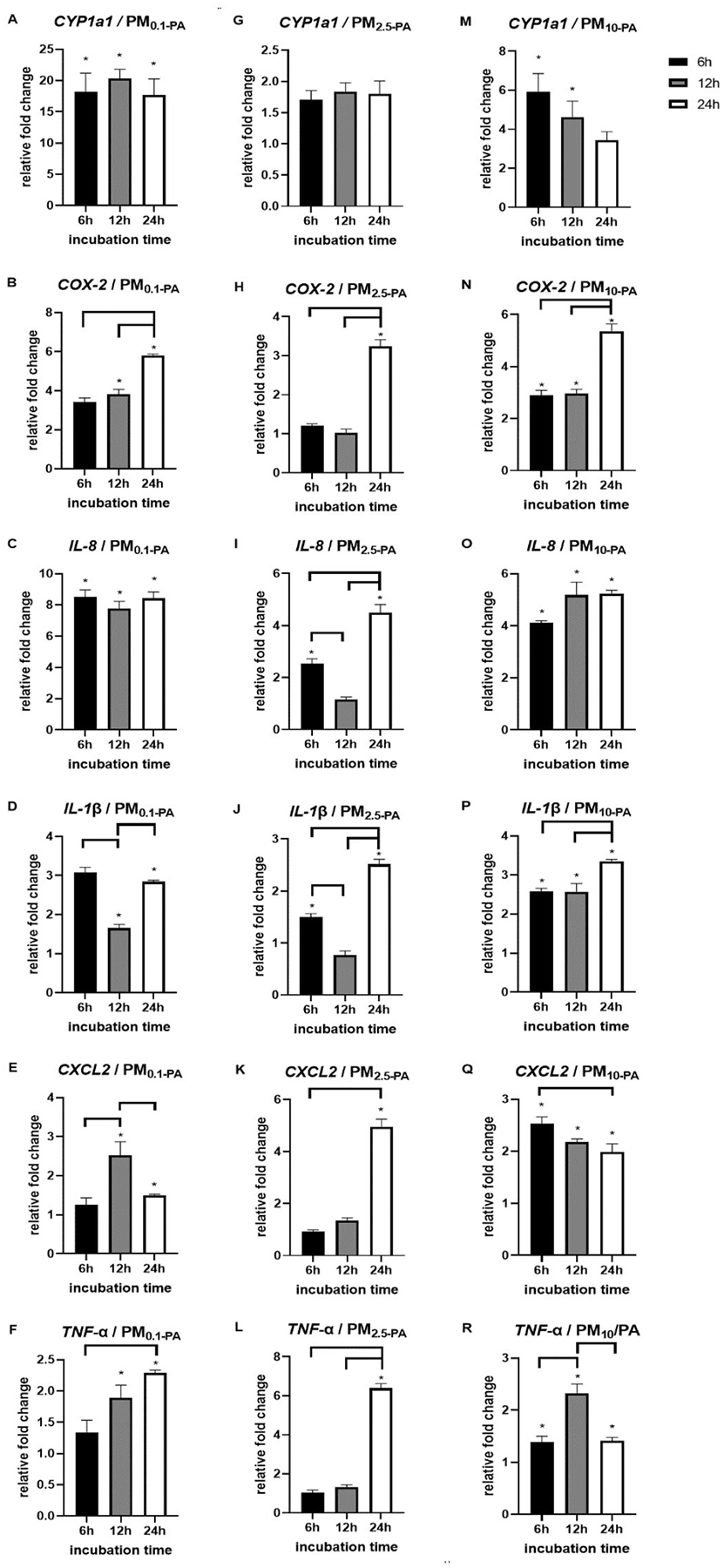
Gene expression profile in U937 macrophages after variable-length exposures to PM_PA_ versus PBS. Gene expression was measured, normalized to that of the β-actin housekeeping gene, and graphed as fold changes relative to the PBS control. Fold changes are shown on the y-axes as functions of 6-, 12-, or 24-h exposure to different size fractions of particulate matter (PM) extracts from San Joaquin Valley (Parlier) (PA), California. Panels A–F, G–I, and M–R represent exposure to PM_0.1-PA_, PM_2.5-PA_, and PM_10-PA_, respectively. Statistical analyses were performed using one-way ANOVAs and post hoc Tukey’s tests. Asterisks (*) denote statistically significant (*p* < 0.05) differences from PBS exposure. Brackets indicate statistically significant (*p* < 0.05) differences between size fractions from the same source.

Further analysis of PM_PA_ revealed different inflammatory effects depending on the duration of exposure. This finding was observed for *COX-2* (Figure 7(B,H,N)), *IL-8* (Figure 7(C,I,O)), *IL-1*β (Figure 7(D,J,P)), *CXCL2* (Figure 7(E,K,Q)), and *TNF-*α (Figure 7(F,I,R)). Positive (e.g.; Figure 7(B)), negative (e.g.; Figure 7(M)), and U- or inverse U- shaped response curves (Figure 7(D,E,I,J,R)) were observed between gene expression and incubation time. The trends were not consistent for each of the measured genes, as PM_PA_ size also appeared to influence the responses. *IL-1*β had a U-shaped trend with exposure to PM_0.1-PA_ or PM_2.5_-_PA_ (Figure 7(D,J)), but not PM_10-PA_. In contrast, *CXCL2* demonstrated three different temporal trends based on particle size. An inverted U trend with PM_0.1-PA_ exposure, a positive trend with PM_2.5-PA_ exposure, and a negative trend with PM_10-PA_ exposure were found. Only *COX-2* exhibited a consistent association between exposure duration and gene expression across all particle types.

### Role of AhR and NF-κB in PM-mediated effects on gene expression

**Figure 8.**
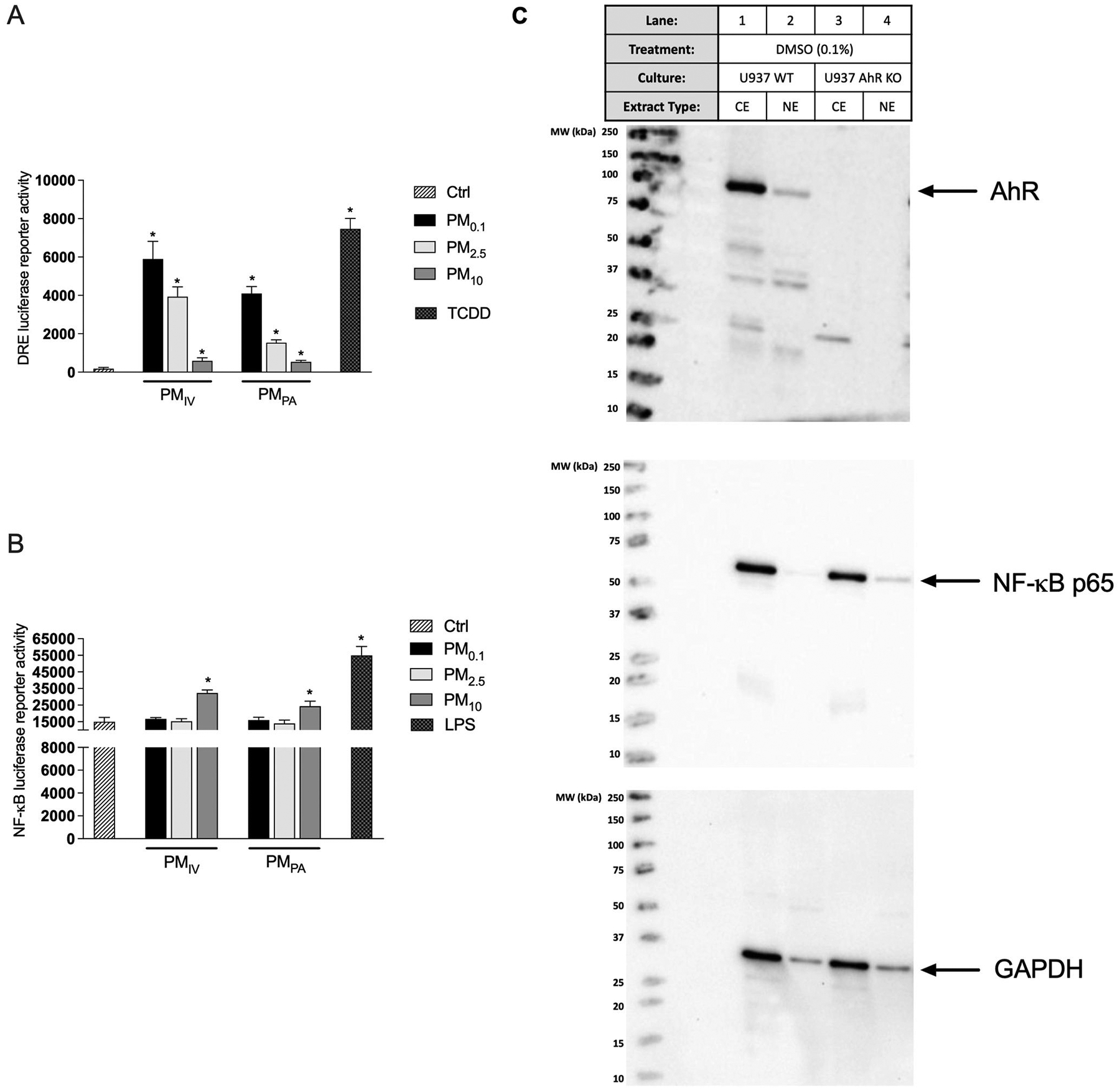
PM induced DRE and NF-κB luciferase reporter activity in HepG2 cells. The cells were incubated for 6 h with 10 μL of PBS negative control (indicated by ‘ctrl’ on the graph); 10 μg/mL stock extracts of ultrafine, fine, or coarse particulate matter (PM_0.1_, PM_2.5_, or PM_10_, respectively) from imperial Valley (IV) or san Joaquin Valley (Parlier) (PA), California; LPS (10 ng/ml) and TCDD (1 nM) were used as positive controls. The graph shows the mean ± standard error of the mean (SEM) for each treatment. The effects of the treatments on (A) AhR activity and (B) NF- κB activity are shown as relative light units from the dioxin-responsive element (DRE) and NF- κB luciferase reporter assay. Asterisks (*) denote statistically significant (*p* < 0.05) increases relative to PBS. (C) Western blot images showing basal protein expression of AhR and NF- κB p65 in cytosol and nuclear fractions of U937 macrophages.

Depending on the source and composition, PM exposure has been found to be associated with increased activity of the AhR and NF-κB signaling pathways (Kanakidou et al. 2005; Vogel et al. 2020). Here we found that exposure to ultrafine PM_IV_ and PM_PA_ for 6 h significantly (*p* < 0.05) increased AhR activity compared to control in cells by 33-fold and 23-fold, respectively (Figure 8(A)). A significant increase of AhR activity was also found for PM_2.5-IV_ and PM_2.5-PA_. Of the PM_IV_ samples, AhR activation potency of the ultrafine PM_0.1-IV_ was the highest followed by increased AhR activity levels induced by PM_2.5-IV_ and PM_10-IV._ This latter pattern was similar for PM_PA_, with the highest AhR activity after treatment with ultrafine PM_0.1-PA_ compared to the two other size fractions (PM_2.5-PA_ and PM_10-PA_). The AhR activation responses to PM_0.1-IV_ were approximately 80% of that to TCDD. TCDD is the prototype of AhR ligands and was used as a positive control. Additionally, we tested if PM exposure would induce the activity of NF-κB. Treatment with the ultrafine PM samples PM_0.1-IV_ and PM_0.1-PA_ and PM_2.5-IV_ and PM_2.5-PA_ did not change the activity of NF-κB (Figure 8(B)). However, treatment with PM_10-IV_ and PM_10-PA_ increased NF-κB activity. The TLR4 ligand LPS was used as positive control.

Expression of AhR and NF-κB p65 protein has been detected using Western blot analysis (Figure 8(C)). Western blot analysis shows the expression of AhR and NFkB p65 protein in the cytosol and at a lower level in nuclear fraction of U937 macrophages in Figure 8(C). AhR protein was not detected in U937 AHR knockout cells. GAPDH was used a housekeeping gene.

### PM-mediated effects on gene expression in AhR-knockout U937 macrophages

**Figure 9.**
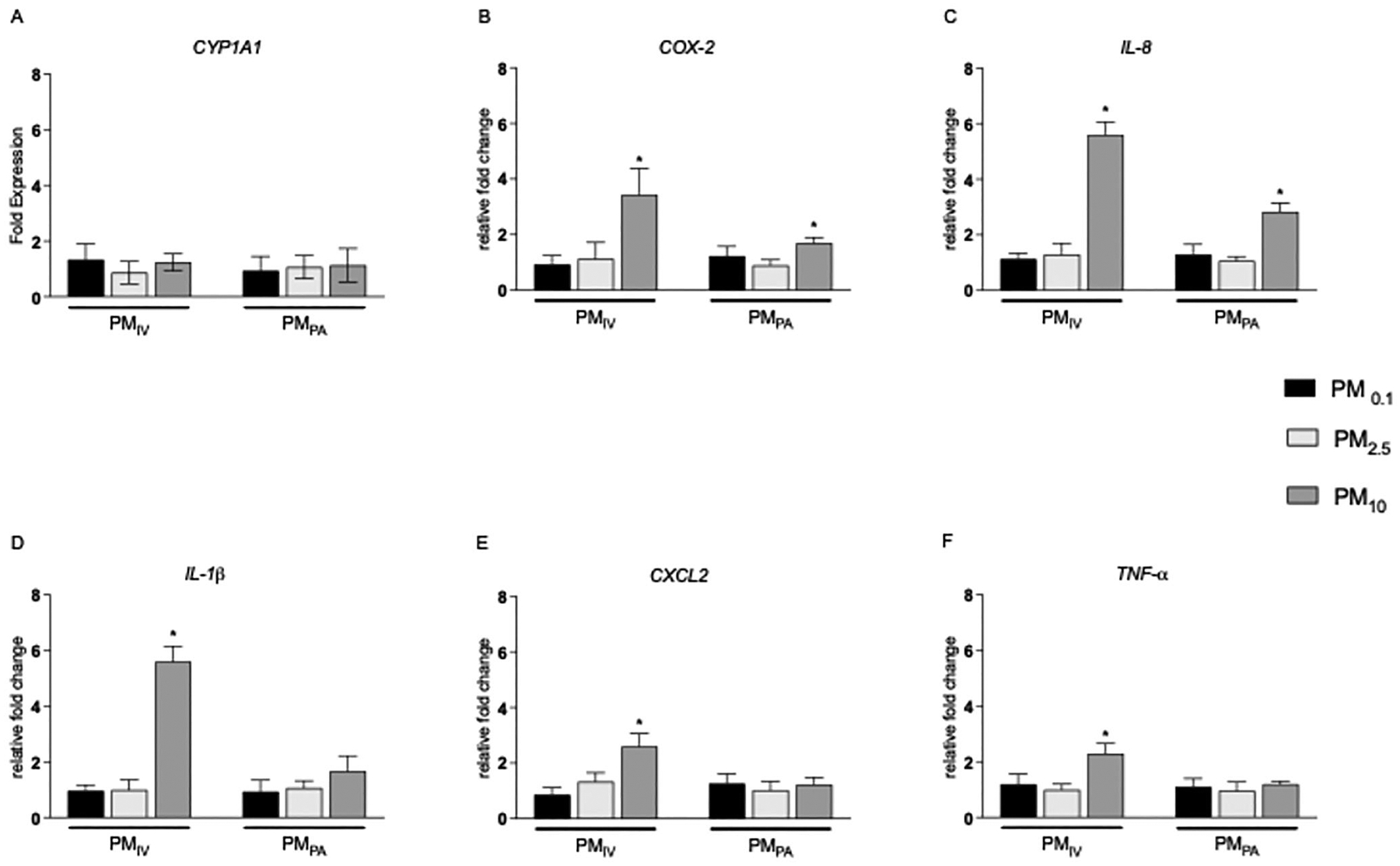
Gene expression profile in PM-treated AhR-knockout U937 macrophages. Gene expression was analyzed and normalized to β-actin 24 h after treatment with PM. Expression of mRNA is shown as fold changes relative to PBS control. Fold changes are shown on the y-axes as functions of exposure to different size fractions of PM from Imperial Valley (PM_IV_) and san Joaquin Valley (Parlier) (PM_PA_). Statistical analyses were performed using one-way ANOVAs and post hoc Tukey’s tests. Asterisks (*) denote statistically significant (*p* < 0.05) differences from PBS exposure.

Based on the finding that PM samples collected in Imperial Valley and San Joaquin Valley (Parlier) significantly increased AhR activity, we tested the role of AhR in mediating the effect of PM samples on mRNA expression of *CYP1a1*, *COX-2*, *IL-8*, *IL-1β*, *CXCL2 TNF-α* in AhR CRISPR knockout U937 macrophages at 24 h after PM treatment. The expression profile of all markers tested revealed that the ultrafine PM_0.1_ and PM_2.5_ samples collected in Imperial Valley or San Joaquin Valley had no significant effect on expression of *CYP1a1*, *COX-2*, *IL-8*, *IL-1β*, *CXCL2* or *TNF-α* in AhR-knockout U937 macrophages (Figure 9(A–F)). However, treatment with PM_10-IV_ significantly increased the expression of *COX-2*, *IL-8*, *IL-1β*, *CXCL2* and *TNF-α* (Figure 9(B–F)). The treatment with PM_10-PA_ led to a small but statistically significant elevated level of *COX-2* and *IL-8* (Figure 9(B,C)).

## Discussion

### Differences due to PM source

Agricultural production is an important source of atmospheric PM, especially in California with the extensive use of dry farming methods (Saxton 1996). The health effects of agricultural PM depend largely on the scale of the operation and regional farming practices that affect PM generation (Pattey and Qiu 2012). Although the composition of ambient PM in the agricultural setting can be highly complex, organic aerosols typically constitute a major constituent of the atmospheric PM (Ishihara et al. 2021). There is increasing evidence the chemical composition of PM plays a vital role in triggering adverse health effects (Plummer et al. 2015; Dreher 2000; Valavanidis et al. 2008) and may explain differences in particle toxicity (Happo et al. 2013).

The findings from this study demonstrate differences in the chemical components of each PM extract. In this study, organic fractions were composed of numerous carbon-containing fragment ions, each with the potential to exert PM toxicity (Sun et al. 2017). A previous study showed that organic compounds in PM can be strongly associated with inflammatory responses (Happo et al. 2010). PM may also contain AhR-activating chemicals, such as dibenzodioxins, dibenzofurans, and non-adjacent substituted polychlorinated biphenyls (PCBs) (Aristizábal et al. 2011). We found that the organic content in PM_IV_ had a much higher percentage than that in PM_PA,_ except for in PM_0.1_ (Figure 1). We speculate a higher proportion of organic content was one of the reasons why PM_IV_ was more potent than PM_PA_ in our gene expression inflammatory biomarker (Figure 5) and AhR activation assay (Figure 8).

High levels of sulfide hydrocarbon organics were also detected in PM_IV_ compared to PM_PA_ (Figure 2). Heavy coal combustion activities in Mexicali, along with the drying lakebed of the Salton Sea may have contributed to the high levels of sulfide hydrocarbon organics identified in PM_IV_. Mendoza et al. (2010) found high levels of sulfate components in PM collected in Imperial Valley consistent with the experimental results from the current study. Sulfur use in agricultural operations may have also contributed to the organic sulfur content in Imperial Valley PM extracts. Therefore, we would speculate that another reason for the higher toxicity of PM_IV_ versus PM_PA_ (Figure 5) may be due to the higher sulfate levels compared to PM_PA_.

Chow and Watson (2001) noted PM_10_ from Mexico produced the most notable health effects at the northern portion of the border area. This effect was only markedly reduced beyond 15 km (Chow and Watson 2001), and Calipatria, the PM sampling site in the Imperial Valley, is approximately 19 km from the Mexican border. Taken together, this information suggests vehicle exhaust or diesel combustion emissions originating at the northern border of Mexico may be transported over long distances into the airshed of the Imperial Valley, thus contributing a proportion of the collected PM_IV_ fraction. However, larger PM fractions are more significantly affected by gravitational forces during transport, thus limiting their transport distance.

Given PM samples from the Imperial Valley and the San Joaquin Valley were collected in summer and fall, respectively, seasonal shifts could have contributed to the varied chemical composition of PM_IV_ compared to PM_PA_. Differences in agricultural practices, wind speed, temperature, and traffic conditions may contribute to seasonal shifts in PM composition. Previous studies have demonstrated that seasonal variations can impact the toxicity of PM (Nawrot et al. 2007), and outdoor particles collected during warmer seasons from the same site were more likely to induce inflammation and cytotoxicity (Happo et al. 2013). Even though PM_IV_ was collected during the summer, season alone cannot explain the stronger inflammatory potential of PM_IV_ versus PM_PA_.

NO_3_^−^ and NH_4_^+^, for example, are influential drivers of PM toxicity (Zhang et al. 2018). A previous study indicated that atmospheric NO_3_^−^ in California peaked in the winter and fall, which was positively correlated with NH_4_^+^ (Bell et al. 2007). NO_3_^−^, NH _4_^+^, and other harmful components (e.g. elemental carbon, organic carbon matter, and sulfate) may increase in fall with residential usage of electric appliances (e.g. heaters) and intensified crop-harvesting practices. Given fall PM_PA_ contained similar or higher NO_3_^−^, and much higher NH_4_^+^ levels (Figure 1) but was less inflammatory relative to summer PM_IV_, summer PM_PA_ could be the least potent of the three extracts due to seasonal declines in atmospheric NO_3_^−^ and NH_4_^+^. The previously reported (Becker et al. 2005) seasonal variations in PM components were based upon samples from the same region. Thus, it is difficult to apply the reported seasonal trends to the current study. However, it is reasonable to speculate that the differences in the chemical compositions of PM_IV_ and PM_PA_ were less impacted by seasonal variation than regional differences such as their geology, geography, agricultural practices, and traffic control.

Establishing a quantitative relationship between specific PM constituents and toxicity/inflammatory response is an important next step. Future studies will incorporate comprehensive chemical profiling coupled with multivariate or regression-based approaches to better resolve the contribution of individual components and their interactions to the observed effects.

### Differences due to PM size

The biological responses to PM_10-IV_ were found to be significantly (*p* < 0.05) higher than that observed for either of the other two size fractions (PM_2.5-IV_ and PM_0.1-IV_) (Figure 5(B–F)). Previous studies have shown that oxygenated organic compounds in PM are strongly associated with inflammatory responses (Plummer et al. 2015; Niu et al. 2021). A higher proportion of oxygenated hydrocarbons may explain the greater effects on inflammatory gene expression of PM_10-IV_ compared to PM_0.1-IV_ and PM_2.5-IV_.

Responses to PM_0.1-IV_ were often significantly (*p* < 0.05) higher than that observed for PM_2.5-IV_ (Figure 5(B–D)), but only occasionally significantly greater when compared to PM_10-IV_ (Figure 5(A)). The sulfate content of secondary inorganic components was highest in PM_0.1-IV_ (Figure 1). Proximity to the Mexican border may account for some of the sulfate content of PM_0.1-IV_ since other inorganic components, such as NH_4_^+^, NO_3_^−^, and chloride, would more easily be depleted during transport (Lanz et al. 2010). Particles generated by sulfur usage in agricultural operations and organosulfates from Salton Sea in the Imperial Valley may be another noteworthy contributor to the formation of ultrafine sulfate.

AhR activation potency of PM_0.1-PA_ was 2.5-fold higher than that of the PM2.5-_PA_ fraction (Figure 8). Though PM_10-PA_ produced similarly strong mRNA responses relative to PM_2.5-PA_ (Figure 5), PM_10-PA_ consisted of primarily organic compounds, unlike PM_0.1-PA_ and PM_2.5-PA_, and it had a relatively high NH_4_^+^ content (Figure 1(D–F)). In a previous study, Meng et al. (2010) found the dominant sources of PM_10_ from the San Joaquin Valley were mobile and stationary fire sources, wood combustion, and ammonium nitrate (NH_4_NO_3_). Another study showed high concentrations of airborne PM in the San Joaquin Valley consisted mainly of NH_4_NO_3_, organic carbon, and elemental carbon, which were thought to be the primary drivers of inflammatory changes in the airways of healthy adult rats (Smith et al. 2003). Cumulatively, results from the two studies (Plummer et al. 2015; Dreher 2000) support our findings, and we presume the higher inflammatory response to PM_10-PA_ was due to its organic matter and NH_4_^+^ salt content. It is worth emphasizing that PM exposure in the present study had no statistically significant effect on the viability of macrophages irrespective of the PM source or size fraction, suggesting the observed biological responses were not due to cell death.

Numerous experiments and epidemiological studies have shown that PAHs are major components of ambient PM, and PAH-induced toxic responses are mediated at least in part by activation of the AhR (Vogel et al. 2020; Korashy and El-Kadi 2006). Identifying the activation effects of the AhR is critical to understanding the physiological and pathological effects of PM exposure. PM_0.1-IV_, PM_2.5-IV_, and PM_10-IV_ all activated AhR (Figure 8), which regulates the expression of *CYP1a1*, Cytochrome P450 Family 1 Subfamily A Member 2 (*CYP1a2*), and Cytochrome P450 Family 1 Subfamily B Member 1 (*CYP1b1*) *via* the canonical AhR pathway, and indirectly, *via* a downstream cascade response and non-canonical AhR pathways, activates the inflammatory markers *COX-2*, *IL-8*, and *IL-1*β (D’Evelyn et al. 2021; Vogel et al. 2007; Kanakidou et al. 2005; Vogel et al. 2020; Attafi et al. 2020; Qi et al. 2020). Based on the results of the DRE luciferase reporter assay (Figure 8), AhR activation by PM_0.1-IV_ was optimal, suggesting that, of all the PM_IV_ fractions, PM_0.1-IV_ may contain the most PAHs. PAHs and dioxin-like chemicals are more likely than other organic compounds to bind to the AhR in the cytoplasm, thus increasing *CYP1a1* expression. Particles of smaller sizes have a higher PAH content because they have higher specific areas and organic compound attachment rates than larger particles. This supports our findings that PM_0.1-IV_ had generally a greater effect on expression of inflammatory markers than PM_2.5-IV_ and sometimes greater effects than PM^10-IV^ (Figure 5). Although PM_10-IV_ significantly increased AhR activation relative to the control, the magnitude of activation was significantly less than that produced by PM_0.1-IV_ and PM_2.5-IV_ (Figure 8). These results suggested that PM_10-IV_-induced *COX-2*, *IL-8*, *IL-1β*, *CXCL2*, and *TNF-α* expression may not be due to the AhR-activating chemical fraction, but rather to the activation of other pathways after internalization in the cell. This is supported by the finding that only the PM_10_ samples induced inflammatory markers in AhR knockout macrophages. Furthermore, the nuclear factor erythroid 2-related factor 2 (Nrf2), AhR, and NF-κB pathways have been found to be activated early after PM_10_ exposure as a compensatory stress mechanism and allows for the possibility that PM_10_ components, especially in the case of PM_10-IV_, act directly as ligands to activate upstream membrane-bound receptors (Radan et al. 2019; Cen et al. 2020; Vogel et al. 2009; Aung et al. 2011; Lamas et al. 2018). NF-κB acts as a key mediator of the inflammatory response regulating the survival, activation, and differentiation of innate immune cells and inflammatory T cells (Churg et al. 2005; Vogel et al. 2021).

Of the PM samples, the PM_0.1_ and PM_2.5_ had a greater effect on AhR activation than PM_10_ (Figure 8). AhR activation was also observed upon exposure to PM_2.5_ in a mouse model studied by Castañeda et al. (2018). This may be due to exogenous AhR ligands with high affinities, such as halogenated aromatic hydrocarbons (HAHs) and PAHs (Denison et al. 1998) in PM_2.5-PA_ relative to PM_0.1-PA_. TCDD, an HAH and potent activator of AhR, is a by-product of biomass combustion and industrial production that can have toxic effects on the host with only trace picomolar amounts (Poland and Knutson 1982). However, the main source of PM_2.5-PA_ is likely not biomass burning. A recent study showed that PM_2.5_ concentration in the San Joaquin Valley (Parlier) region is highest in winter and lowest in summer (Watson et al. 2021). The study reported that seasonal concentration variability, and annual PM_2.5_ concentrations in general, decreased substantially from 2014 to 2019 due to a series of stringent local government measures to limit industrial emissions but have stabilized in recent years.

### Differences due to exposure duration

Previous *in vitro* studies have consistently shown that exposure to PM_2.5_ affects inflammatory responses in a time-dependent manner (Deng et al. 2013; Abbas et al. 2019). This is fairly consistent with the current experimental results. Short-term (6-h, 12-h, and 24-h) PM_2.5_ and PM_0.1_ *in vitro* exposures resulted in strong positive correlations between the intensity of the inflammatory response and the duration of exposure (Figure 6(A–I), Figure 7(A–I)). In contrast, PM_10_ exposure resulted in negative correlations between the expression of genes (*CXCL2* and *TNF-α*) and exposure duration (Figure 6(Q,R)). The influence of exposure duration was observed with both PM_IV_ and PM_PA_.

A plausible reason for the observed time-dependent responses could be that PM_2.5_ exposure increases the expression of proinflammatory cytokines (e.g. *TNF-α*) (Panasevich et al. 2009). Prolonged exposure to PM_2.5_ could lead to greater oxidative stress and prolonged activation of cellular- and cytokine-mediated pathways, including apoptosis, which could progress to a chronic inflammatory response with insufficient clearance of apoptotic cells (Grabiec and Hussell 2016; Robb et al. 2016).

PM_10_ exposure resulted in variable levels of *TNF-α* expression with increasing duration, albeit to a limited extent (Figure 5(r) and 6(r)), implying that the magnitude of change in *TNF-α* due to cellular oxidative stress may be masked by high degeneration. In the American MESA study, Diez-Roux et al. (Diez Roux et al. 2006) found no consistent evidence of a positive correlation between short-term exposure (24 h to 48 h) to PM_10_ and c-reactive protein concentrations. In the inflammation investigation in the Tel-Aviv Metropolitan area in Israel, the relationship between short-term (4-day) exposure to PM_10_ and the inflammatory response was also found to be uncorrelated (Steinvil et al. 2008). In the PM_10_ pollutant model, the test population did not show any statistically significant association with the high-sensitivity c-reactive protein (hs-CRP) or total white blood cell count inflammatory biomarkers. Similarly, Tsai found that short-term (1-day or 1-week) exposure to PM_10_ was uncorrelated with *TNF-*α levels in men (Tsai et al. 2019). Findings from these three studies are consistent with the results of the present study (Diez Roux et al. 2006; Steinvil et al. 2008; Tsai et al. 2019). However, the absence of correlation between PM_10_ and *TNF-*α does not indicate that PM_10_ is devoid of inflammatory potential. On the contrary, *TNF-*α, as a marker of inflammatory response, may not be suitable for detecting acute respiratory inflammation caused by PM_10_.

The U- and inverse U-shaped response patterns observed in the mRNA expression levels of many of the inflammatory biomarkers could be attributed to the intrinsic chemical and size differences of the PM extracts. This is supported in part by significant variations in chemical composition (e.g. oxidative organics and metal content) between and among the PM_IV_ and PM_PA_ fractions. U- and inverted U-shaped dose responses may be common due to adaptive effects or accumulation of the active compound. It is possible that 1) the U-shaped responses observed in this study were a result of transient immunosuppression and recovery after the 12- and 24-h exposures, respectively; and 2) the inverted U-shaped responses resulted from the stabilization of rapidly increasing responses. However, the mechanisms driving the response trends were not clearly established in the present study but should be a topic of future research.

### Role of AhR and NF-κB in PM-mediated effects on gene expression

The results from luciferase assay and PCR analysis showed that ultrafine PM_0.1_ and PM_2.5_ samples from Imperial Valley and San Joaquin Valley significantly induced the activity of AhR and the expression of *CYP1a1* and the selected inflammatory markers. In contrast to wild type U937 macrophages, PM_0.1_ and PM_2.5_ samples did not change the expression of *CYP1a1* or inflammatory markers in AhR knockout macrophages. On the other hand, PM_10-IV_ significantly increased inflammatory markers *COX-2*, *IL-8*, *IL-1β*, *CXCL2,* and *TNF-α* in AhR knockout macrophages. The results suggest that the induction of *CYP1a1* and inflammatory genes by PM_0.1_ and PM_2.5_ from Imperial Valley and the San Joaquin Valley are mediated *via* AhR-dependent signaling. However, the increased levels of the inflammatory markers induced by PM_10_ seem to be AhR-independent and may involve other signaling pathways such as NF-κB. Interestingly, previous reports have shown that PM induced inflammatory markers and CYP1A1 in lung BEAS-2B cells, keratinocytes as well as in healthy young adults also (Tripathi et al. 2018, Jang et al. 2019, Yang et al. 2022).

The data demonstrate that ultrafine and fine PM preferentially engage AhR-dependent signaling, whereas coarse PM activates NF-κB–mediated inflammatory pathways, highlighting size-fraction–specific modes of action.

### Study limitations and suggestions for future research

The main goal of this study was to qualitatively investigate the biological responses of exposure to different size fractions of PM that contain different chemical compositions and come from different sources. Therefore, these three factors were analyzed independently. However, the interactive effects between PM source, size fraction, and exposure duration were not statistically investigated using a multivariate analysis of variance. The results are therefore subject to potential effect modification. We detected positive time-dependent effects for PM_0.1_ and PM_2.5_ (Figure 6, Figure 7), with unique inflammatory responses for each size fraction when incubated for the same time (Figure 5). Dose–response analyses are planned for future work and may give us additional insight into the biological responses of PM. The gene panel was designed to focus on early, robust pro-inflammatory and xenobiotic-responsive pathways as part of a screening strategy to compare PM samples under controlled conditions. As such, we prioritized markers that are well established to respond acutely to PM and PAH exposure. However, we acknowledge that this approach does not capture the full spectrum of macrophage functional states, particularly those relevant to type 2 inflammation and tissue remodeling, which are relevant in the context of allergic airway disease. Furthermore, we conducted a chemical composition analysis, which may explain the different responses (Figures 1 and 2). The results also suggest that the effects for PM_0.1_ and PM_2.5_ on gene expression are AhR-dependent (Figure 9). While our findings may be conservative and underestimate the synergistic effects of source, size, exposure time, and composition, they revealed trends in association and notable biological responses.

A second limitation is the lack of *in vivo* experiments. We recognize that the macrophages as a monoculture model does not fully capture the complexity of the pulmonary environment, but they enable controlled, particle-specific comparisons. Our *in vitro* models were unable to mimic the complexity of the biological milieu and the human immune response. Therefore, our estimates of inflammatory biomarker expression levels may be overstated. However, our findings provided significant insights into the source-, size-, and exposure-specific PM-induced inflammation that can occur *in vivo*, with specific gene targets that could be used for mechanistic experiments. Our findings could also be used to support a future comparative analysis of *in vitro* and *in vivo* responses.

Future studies should include advanced statistical analyses of the direct and interactive effects of PM source, size, exposure duration, and dose–response analyses related to induced inflammatory and AhR-mediated responses *in vitro* and *in vivo*. Animal experiments should be conducted for *in vivo* validation of the trends we observed in macrophages.

## Conclusions

The findings of the present study suggest (1) PM_IV_ induces greater expression of inflammatory genes compared to size-matched PM_PA_; (2) ultrafine and coarse particles stimulate inflammatory gene expression primarily after exposure for 12-h irrespective of the PM source; 3) despite the absence of a direct correlation between gene expression and exposure duration, 24-h exposures to ultrafine PM_IV_ or fine PM_IV_/PM_PA_ produced similar effects; and (4) ultrafine PM_IV_/PM_PA_ were the most potent AhR activators. This study supports previous findings regarding the effects of PM on human health. This is the first study to compare and elucidate the effects of PM sources, as well as different sizes, and exposure durations from the same source on inflammatory gene expression and AhR activation. Thus, this study provides new insights to better understand the harmful effects from PM exposure on human health.

## Supplementary Material

Supp 1

Supplemental data for this article can be accessed online at https://doi.org/10.1080/08958378.2026.2676722.

## Data Availability

The datasets during and/or analyzed during the current study available from the corresponding author on reasonable request.
