# Supplementary material for: Regional and size-dependent effects of ambient agricultural particulate matter on Ah-receptor activity and inflammatory responses in human U937 macrophages
Source: Inhal Toxicol. Author manuscript; Available in PMC 2026 Jun 23. (PMC13286247; doi:10.1080/08958378.2026.2676722)
Supplement: Supp 1 [file NIHMS2185030-supplement-Supp_1.docx]

## 8. SUPPLEMENTAry FIGURES


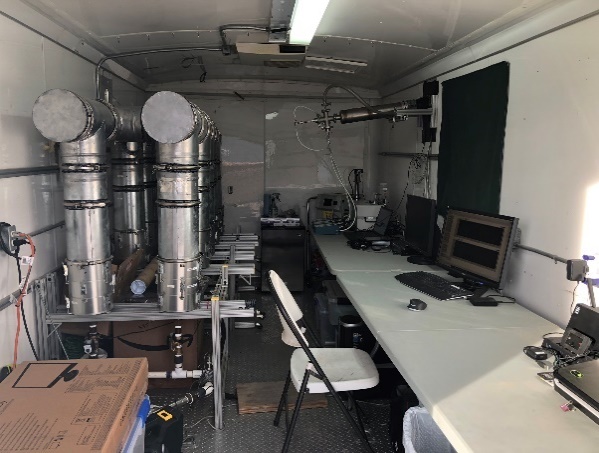

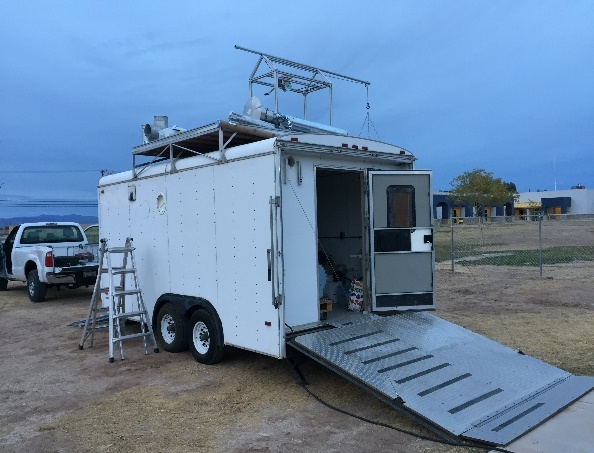


**Supplementary Figure S1.** The fully remote-controllable PM Sampling and Measurement Platform (PMSAMP) use for PM collection. The left and right panels show the internal and external structures of the PMSAMP.


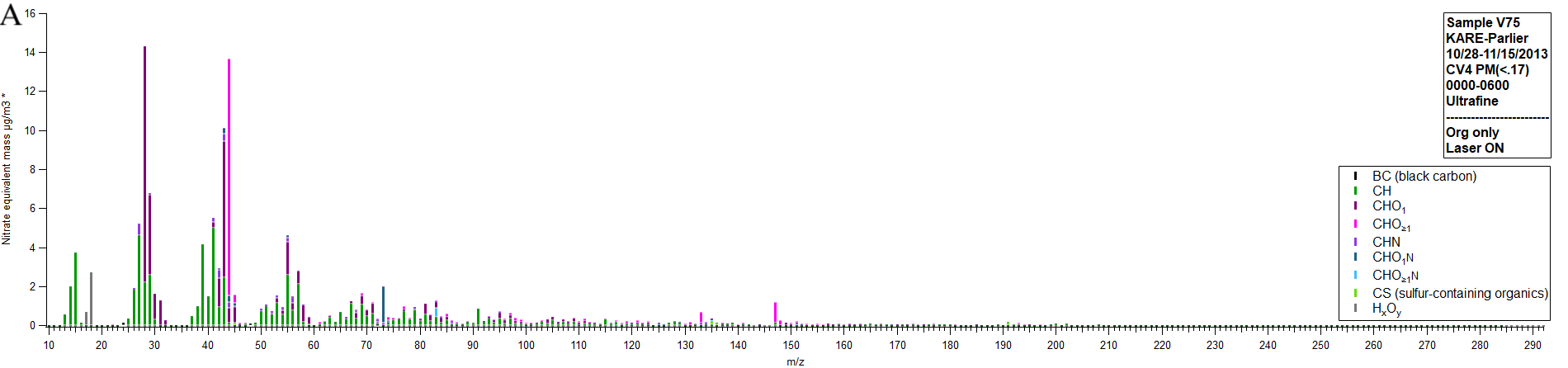


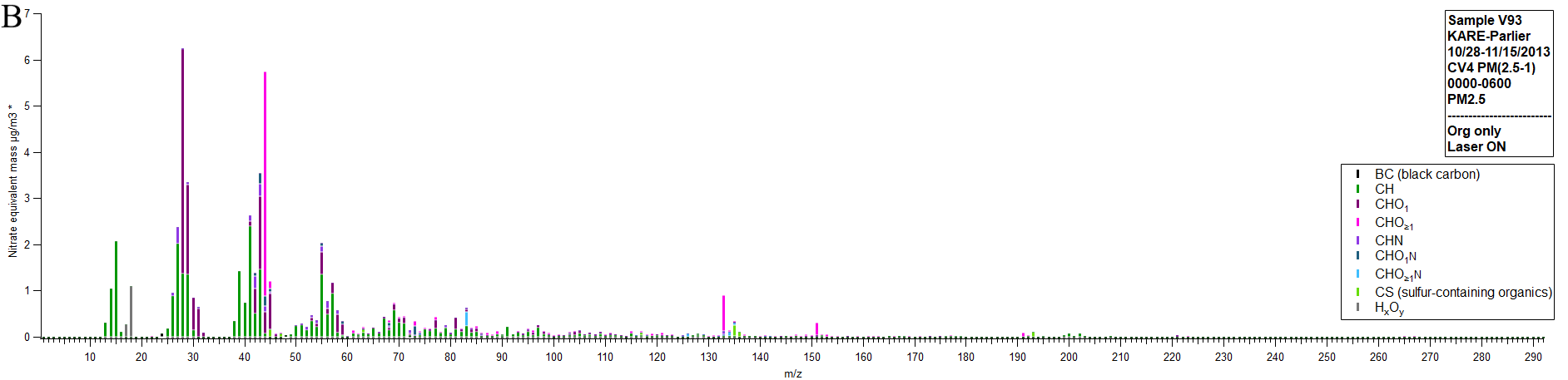


**Supplementary Figure S2.** The Aerosol Mass Spectrometry (AMS) spectra of PM_0.1-PA_ (b) and PM_2.5-PA_ (b).

**Supplementary Figure S3.** Gene expression profile after 12-h incubation of U937 macrophages with PM extracts. Gene expression of the housekeeping genes β-actin, gapdh, and rps13 was measured and graphed as relative changes to control. Fold changes are shown on the y-axes as functions of exposure to different size fractions of particulate matter (PM) extracts from Imperial Valley (IV) and San Joaquin Valley (PA).

Table S1. Fractional NR-PM composition for the PM extracts shown in Figure 1.

| **Category** | **PM_0.1 - IV_** | **PM_2.5 - IV_** | **PM_10- IV_** | **PM_0.1 PA_** | **PM_2.5 PA_** | **PM_10 PA_** |
| --- | --- | --- | --- | --- | --- | --- |
| Organics | 69 | 85 | 81 | 76.5 | 81.7 | 45.4 |
| NO_3_^-^ | 3 | 8 | 11 | 2.1 | 2.9 | 25.7 |
| SO_4_^2-^ | 21 | 2 | 2 | 1 | 0.9 | 4.4 |
| Cl^-^ | 3 | 4 | 5 | 11.9 | 7.7 | 13.8 |
| NH_4_^-^ | 4 | 1 | 1 | 7 | 5.1 | 10.2 |
| Metals | N/A | N/A | N/A | 1.5 | 1.7 | 0.5 |

Table S2. Fractional organic aerosol composition for the PM extracts shown in Figure 2.

| **Category** | **PM_0.1 - IV_** | **PM_2.5 - IV_** | **PM_10- IV_** | **PM_0.1 PA_** | **PM_2.5 PA_** | **PM_10 PA_** |
| --- | --- | --- | --- | --- | --- | --- |
| C_x_H_y_ | 38.5 | 46.7 | 39.2 | 44.8 | 46.2 | 28.4 |
| C_x_H_y_O_1_ | 30.7 | 25.5 | 30.3 | 30.5 | 27.3 | 37.6 |
| C_x_H_y_O_>1_ | 18.9 | 11.9 | 17.2 | 14.8 | 14.4 | 19.9 |
| H_x_O_y_ | 3.4 | 1.7 | 3.5 | 2.6 | 2.4 | 4.2 |
| C_x_H_y_N_p_ | 5.7 | 9.8 | 6.7 | 3.3 | 4.3 | 6.1 |
| C_x_H_y_O_z_N_p_ | 2.3 | 4 | 2.7 | 3.2 | 3.2 | 3.4 |
| C_x_H_y_O_z_S_q_ | 0.5 | 0.4 | 0.4 | 0.8 | 2.2 | 0.4 |
